# The Church, Practitioners, and the Marriage of Syphilitics

**Published:** 1919-07-26

**Authors:** G. Arbour Stephens


### THE EDITOR'S LETTER-BOX.

**[Correspondence on all subjects is invited, but we cannot in any way be responsible for the opinions expressed by our correspondents, who must give their name and address as a guarantee of good faith, but not necessarily for publication. Correspondents are reminded that brevity of style and conciseness of statement greatly facilitate early insertion.]**

### The Church, Practitioners, and the Marriage of Syphilitics.

*To the Editor of* The Hospital.

Sir,—Are you honest in suggesting in your leading article on June 28 that I shrank from taking all the steps possible?

The marriage was by licence, and I informed the registrar who issued the licence.

Late on Friday afternoon I got the information; that evening I informed the priest; the next day (Saturday) I went to the registrar, and the priest married them on Sunday.

The woman died, at the end of nine months, in labour.—

Swansea. G. Arbour Stephens.

[Dr. G. Arbour Stephens seems to have done all that a man could do to prevent the marriage. He should refer to our leader, when he will find we do not state that he shrank from taking all the steps possible.—Ed. "The Hospital."]

